# Femtosecond Laser Fabrication of Anatase TiO_2_ Micro-nanostructures with Chemical Oxidation and Annealing

**DOI:** 10.1038/s41598-017-02369-w

**Published:** 2017-05-18

**Authors:** Ting Huang, Jinlong Lu, Xin Zhang, Rongshi Xiao, Wuxiong Yang, Qiang Wu

**Affiliations:** High-power and Ultrafast Laser Manufacturing Lab, Institute of Laser Engineering, Beijing University of Technology, Beijing, 100124 China

## Abstract

The fabrication of nanoporous anatase TiO_2_ on a microstructured Ti base is achieved through an innovative hybrid fabrication method involving femtosecond laser ablation coupled with H_2_O_2_ oxidation and annealing. The anatase TiO_2_ micro-nanostructures have superior photocatalytic degradation of methyl orange due to enhanced light harvesting capacity and surface area. The photodegradation efficiency increases by a maximum of 80% compared to the nanoporous anatase TiO_2_ fabricated through H_2_O_2_ oxidation and annealing only (without femtosecond laser ablation). Meanwhile, The anatase TiO_2_ micro-nanostructures show good cyclic performance, indicating a great potential for practical application. The proposed hybrid method can easily tune the morphology and size of microstructure by simply adjusting the femtosecond laser parameters, showing advantage in fabricating of micro-nanostructures with a rich variety of morphologies.

## Introduction

Titanium dioxide (TiO_2_) is one of the most important semiconductor materials, substantiated by its wide range of applications including catalysis and energy harvesting/storage^1–5^. The performance of TiO_2_ is affected tremendously by modification of chemical composition or construction of favorable structures^5–7^. Recently, the three-dimensional micro/nanostructured TiO_2_ with enhanced performance have been widely reported in applications such as photocatalysis, dye-sensitized solar cells, and lithium-ion batteries, mainly due to the structure-induced properties including large specific surface area, superior light harvesting capacity, and prominent carrier mobility^8–11^.

The fabrication of micro/nanostructured TiO_2_ for photocatalysis has attracted continued interest from both scientific research and industrial applications^10^. Currently, in many photocatalytic applications, e.g. aqueous purification, micro/nanostructured TiO_2_ is mostly fabricated in the form of suspended powder, which is difficult to recycle from aqueous solution after photocatalytic reactions and will cause secondary pollution. The most common approach to solve this problem is to fabricate self-supported TiO_2_ micro-nanostructures by coating TiO_2_ particles onto a conductive substrate to form a film or membrane, as is the case with fabricating photocatalytic purifiers at present^12, 13^. However, the coated TiO_2_ particles are prone to stripping from the substrate, leading to compromised photocatalytic properties.

Alternatively, bulk-supporting substrate with directly grown TiO_2_ is a choice for eliminating the drawbacks of powder. There are many essential factors for high photocatalytic properties of substrate-grown TiO_2_, such as nanostructures of TiO_2_ and microstructures of supporting substrate^14–18^. There have been several reports related to nanostructured TiO_2_^14–16^. However, there lacks research on structuring the substrate. Typical fabrication methods for structuring the substrate include acid etching^17^, spot welding^18^, etc. The structures of the substrate fabricated by these methods generally are uncontrollable or have limited surface area. Therefore, the development of facile methods for the fabrication of substrate-grown TiO_2_ with designable structure of both nanoscale TiO_2_ and microscale substrate is highly desirable.

**Figure 1:**
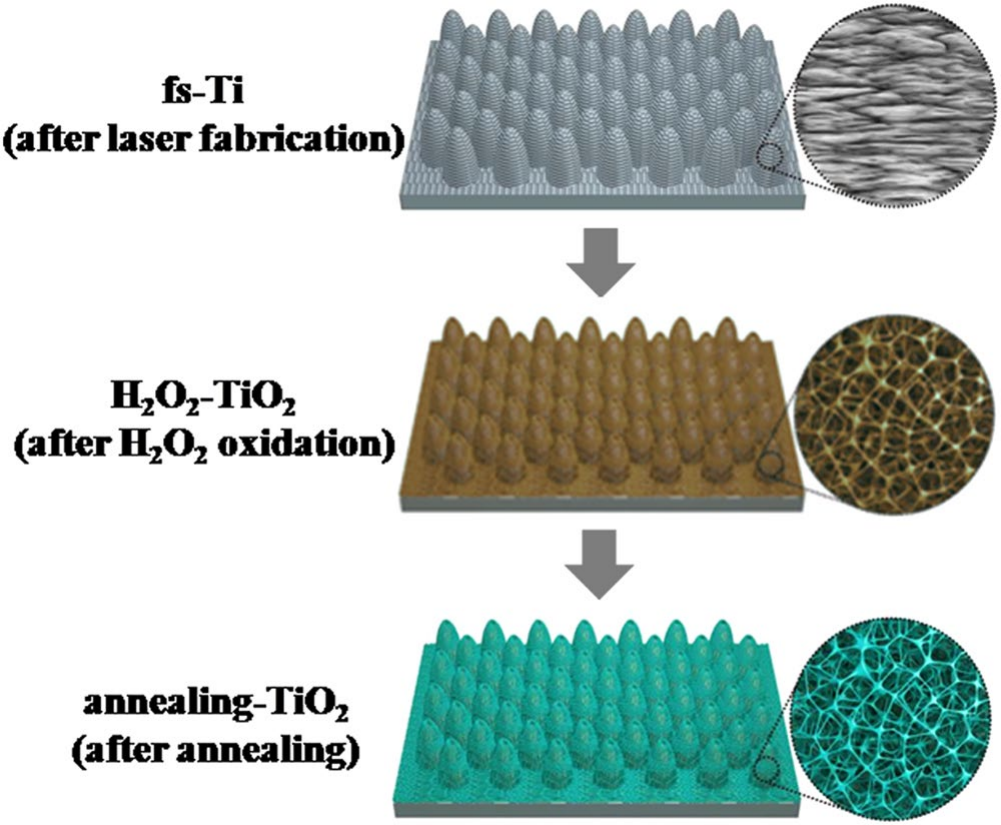
Schematic of the fabrication of anatase TiO_2_ micro-nanostructures on Ti substrate by the laser-chemical-hybrid method (Grey: fs-Ti, Brown: H_2_O_2_-TiO_2_, Green: annealing-TiO_2_).

In this work, we report a hybrid method for non-coating fabrication of substrate-grown TiO_2_ growing directly from Ti substrate. Figure 1 is a schematic of the fabrication process. First, the primary microarray was fabricated on the surface of Ti plate by a femtosecond laser (fs-Ti). Next, the secondary nanoporous structure was grown from the fs-Ti surface by H_2_O_2_ oxidization to form self-supported H_2_O_2_-TiO_2_. Finally, the H_2_O_2_-TiO_2_ was thermally treated to induce anatase TiO_2_ (annealing-TiO_2_) without changing the morphology. The annealing-TiO_2_ can be tailored in microscale (morphology and size) by femotosecond laser to achieve excellent light trapping capacity for a wide range of wavelength with its reflectance below 5% in 200–1000 nm range. In comparison to nanoporous-TiO_2_ fabricated through H_2_O_2_ oxidation and annealing only (without laser fabrication), the annealing-TiO_2_ exhibited improved photocatalytic degradation of methyl orange and showed excellent stability under ultraviolet-visible (UV-vis) light irradiation.

## Results and Discussion

**Figure 2:**
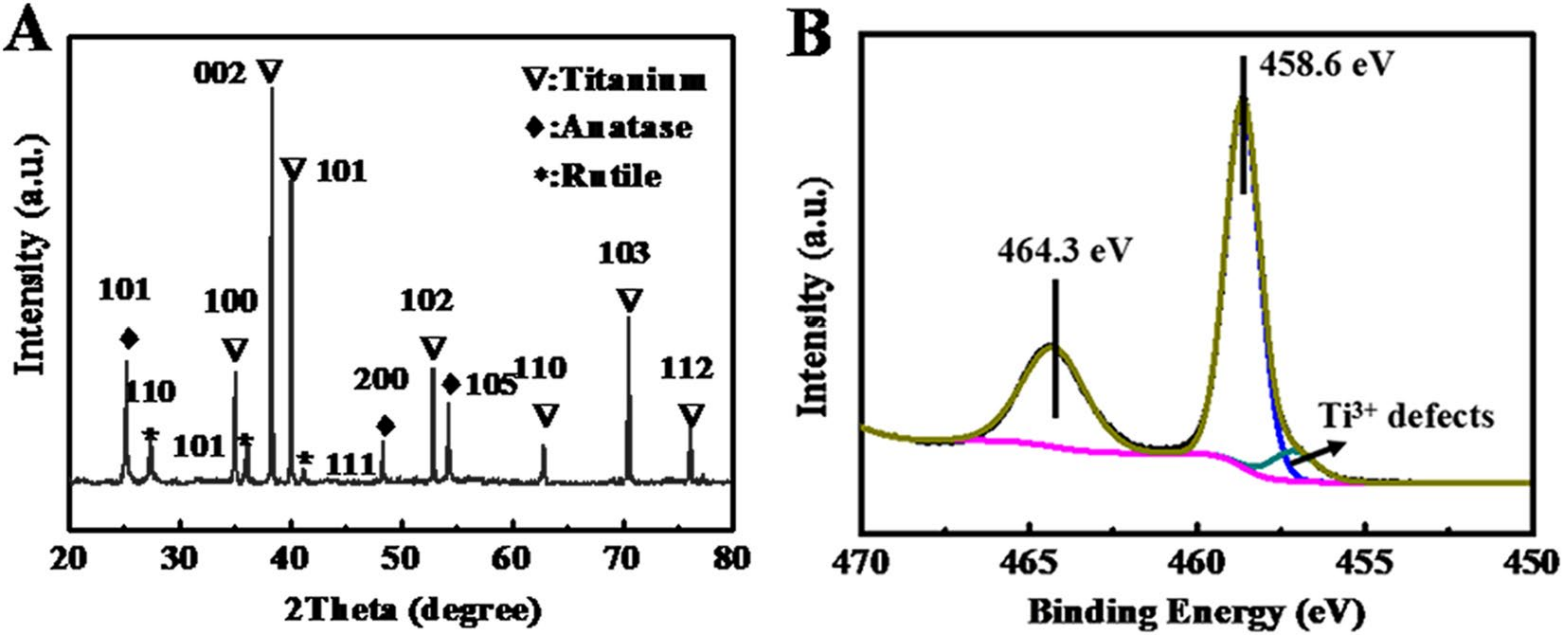
(**A**) XRD pattern and (**B**) Ti 2p XPS of the final structure fabricated by the proposed hybrid method.

Figure 2A shows the X-ray diffraction (XRD) pattern of the final structure (annealing-TiO_2_) fabricated by our hybrid method. Although Ti from the supporting substrate was determined to be the major component, anatase TiO_2_ was clearly identified (JCPDS No. 21–1272). Besides, a little amount of rutile TiO_2_ was also confirmed (JCPDS No. 21–1276). X-ray photoelectron spectroscopy (XPS) was used to further determine the composition. On the surface of the final structure, Ti and O were found to be the main constituents. In the Ti 2p spectrum (Fig. 2B), the peaks of Ti 2p_1/2_ and Ti 2p_3/2_ were centered at 464.3 eV and 458.6 eV with a spin energy separation of 5.7 eV, which is characteristic of TiO_2_, agreeing with previous literature^19, 20^. Furthermore, a 1.5 eV wide shoulder appeared at the right of the Ti 2p_3/2_ peak, indicating Ti^3+^ defects on the surface. It was presumed that the existence of Ti^3+^ defects affected the electron-hole recombination process and thus facilitated the degradation of organics in photocatalytic process^20–22^.

Figure S1A (Supplementary Information) shows XRD patterns of the structure after laser fabrication (fs-Ti) and H_2_O_2_ oxidation (H_2_O_2_-TiO_2_). In the fs-Ti structure, Ti was determined to be the major component with a little amount of TiO. XPS result further confirmed that the fs-Ti structure was composed of Ti, TiO and rutile TiO_2_ (Figure S1B). Previous study also proved the generation of rutile TiO_2_ after femtosecond laser fabrication^23^. The absence of rutile TiO_2_ in XRD result may due to its low amount. After H_2_O_2_ oxidation, unstable TiO disappeard and anatase TiO_2_ formed in the H_2_O_2_-TiO_2_ structure (Figure S1A and C). However, the intensity of XRD peaks of anatase TiO_2_ was relatively low. The transmission electron microscope (TEM) image with diffraction pattern shows most of TiO_2_ formed by H_2_O_2_ oxidation was amorphous (Figure S2), leading to low intensity of anataseTiO_2_. The crystalline of anatase TiO_2_ was improved significantly after annealing identified by increased intensity of diffraction peaks (Fig. 2A). Therefore, the composition change was clear in the hybrid method. The Ti-TiO_2_-anatase TiO_2_ transformation was obtained through laser fabrication, H_2_O_2_ oxidation, and annealing. It has been well known that the ultrashort pulses of femtosecond laser or picosecond laser contribute to the minimization of thermal effect during fabrication^24–29^, maintaining Ti in its original elemental form by minimizing generation of undesirable compounds of Ti, which is necessary for subsequent H_2_O_2_ oxidation process.

**Figure 3:**
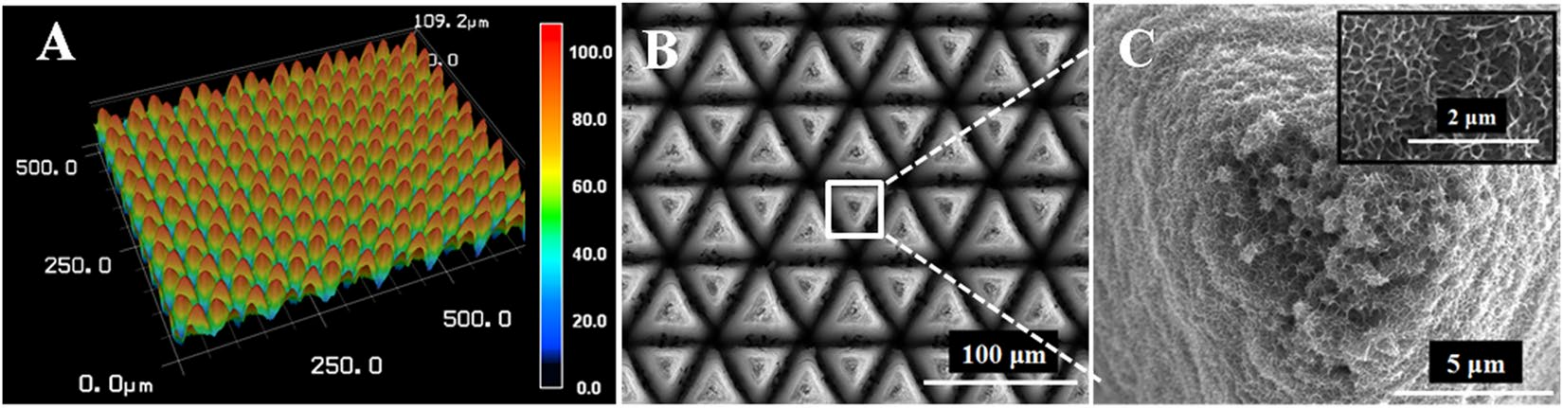
(**A**) LSCM image, (**B**) and (**C**) FESEM top-view images at different magnifications of the final annealing-TiO_2_.

Figure 3A shows the laser scanning confocal microscopy (LSCM) image of the final annealing-TiO_2_. The structure was composed of regular pillars with an average height of 109 μm. The top-view of the field-emission scanning electron microscope (FESEM) shows that these pillars had triangular morphology (Fig. 3B). At higher magnification, the nanoporous substructure can be clearly seen on the surface of the triangle pillars (Fig. 3C and inset). The energy dispersive spectrometer (EDS) result confirmed that the nanoporous structure mainly consisted of titanium and oxygen, a chemical composition that agreed well with the TiO_2_ (Figure S3).

**Figure 4:**
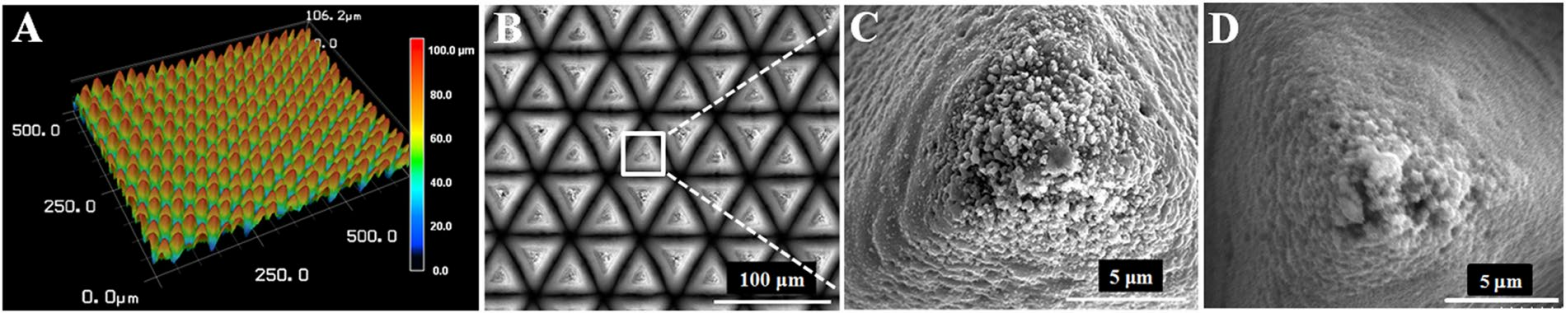
(**A**) LSCM image, (**B**) and (**C**) FESEM top-view images at different magnifications of fs-Ti, and (**D**) SEM top-view image of H_2_O_2_-TiO_2_.

The structure characteristics of annealing-TiO_2_ on microscale were inherited from the laser fabricated fs-Ti, as shown in Fig. 4A and B, respectively. However, the substructures on nanoscale were different. Instead of nanoripples and particles on the surface of laser fabricated fs-Ti (Fig. 4C), nanoporous structure was observed on the surface of annealing-TiO_2_. Figure 4D shows the nanoporous structure was formed in the H_2_O_2_ oxidation process. There was no obvious difference between the morphologies of the nanoporous structure before and after annealing. It has been reported that amorphous TiO_2_ porous film can be obtained via direct H_2_O_2_ oxidation of metallic Ti plate. The basic mechanism for reaction between Ti and H_2_O_2_ has been proposed including dissolution and corrosion of Ti^30, 31^. Initially, Ti(OH)_4_ came into being through oxidation of Ti by H_2_O_2_. And Ti(OH)_4_ was decomposed due to unstable thermodynamics and produced amorphous titania sol. At the same time, the rapid deposition of sol onto the Ti plate surface was disturbed by the dissolution process and porous structure was formed. The results show that the annealing-TiO_2_ has combined micro- and nano-composite features, derived from laser fabrication and H_2_O_2_ oxidation, respectively.

**Figure 5:**
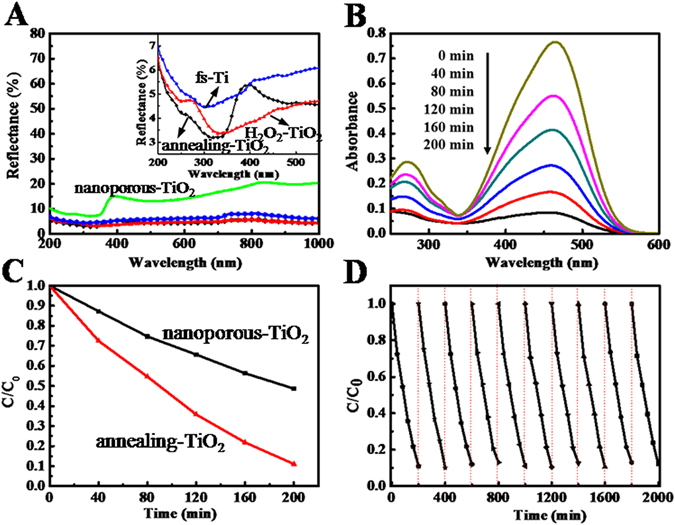
(**A**) Reflection spectra of nanoposours-TiO_2_, fs-Ti, H_2_O_2_-TiO_2_ and annealing-TiO_2_, (**B**) Temporal UV-visible adsorption spectral of annealing-TiO_2_ changes for the methyl orange solution as a function of UV (365 nm) irradiation time, (**C**) Comparison of photocatalytic degradation rates of methyl orange between nanoporous-TiO_2_ structure and annealing-TiO_2_, (**D**) Cycling degradation curve of annealing-TiO_2_.

Figure 5 collectively demonstrates the superior photoanalytic properties of the final annealing-TiO_2_, including its light harvesting capacity (Fig. 5A), photodegradation performance (Fig. 5B and C), and reusability (Fig. 5D). Specifically, Fig. 5A compares the reflectance of the final annealing-TiO_2_ (black line) to that of fs-Ti (after laser fabrication, blue line), and H_2_O_2_-TiO_2_ (after laser fabrication and H_2_O_2_ oxidation, red line), as well as that of the nanoporous-TiO_2_ (with H_2_O_2_ oxidation and annealing but without laser fabrication, green line). The fs-Ti, H_2_O_2_-TiO_2_, and annealing-TiO_2_ all exhibited significantly lower reflectance (3–7%) compared to nanoporous-TiO_2_ (10–20%). This was due to the multi-reflection or scattering of incident light caused by the micro/nano structures created by femtosecond laser, which was also evident in previous studies^26–29^. Since both H_2_O_2_ oxidation and annealing processes preserved the micro feature enabled by initial laser ablation, the desirable low reflectance was thus inherited throughout the entire process. Further, a jump decrease in the reflectance of annealing-TiO_2_ was identified below the 400 nm in wavelength (Fig. 5A inset). This was due to the formation of anatase TiO_2_ with band gap of 3.2 eV, leading to the enhanced UV-light absorption.

Figure 5B shows the temporalvariation of the UV-visible light adsorption spectrum of the methyl orange solution under photocatalysis by annealing-TiO_2_. A major absorption band was clearly identified at 460 nm. The absorption peak underwent a fairly large decrease, i.e. from 0.78 to 0.08, over time, which confirms the effectiveness of the annealing-TiO_2_ in the photodegradationof methyl orange. Figure 5C shows a comparison of photocatalytic degradation rates between annealing-TiO_2_ and nanoporous-TiO_2_. After 200 min irradiation, methyl orange was almost decomposed by annealing-TiO_2_. The photodegradation efficiency increased by 80% compared to the nanoporous-TiO_2_ (90% Vs 50%). The higher photodegradation efficiency is achieved due to higher incident light harvesting capacity and large specific surface provide by micro/nanostructures^21, 32^. Figure 5D shows the cyclic performance of annealing-TiO_2_. The photodegradation of methyl orange was monitored for ten cycles. Each cycle lasted 200 min. After each cycle, the annealing-TiO_2_ was washed and dried thoroughly, then put in the fresh methyl orange solution. The photodegradation rate remained constant over ten consecutive cycles, indicating that the photocatalytic properties of annealing-TiO_2_ were stable and sustained repetitive usage. Figure S4 shows there was no obvious change on the structure features and compositions of the annealing-TiO_2_ after the cycling degradation tests, which further proved that the as-fabricated structure was stable.

**Figure 6:**
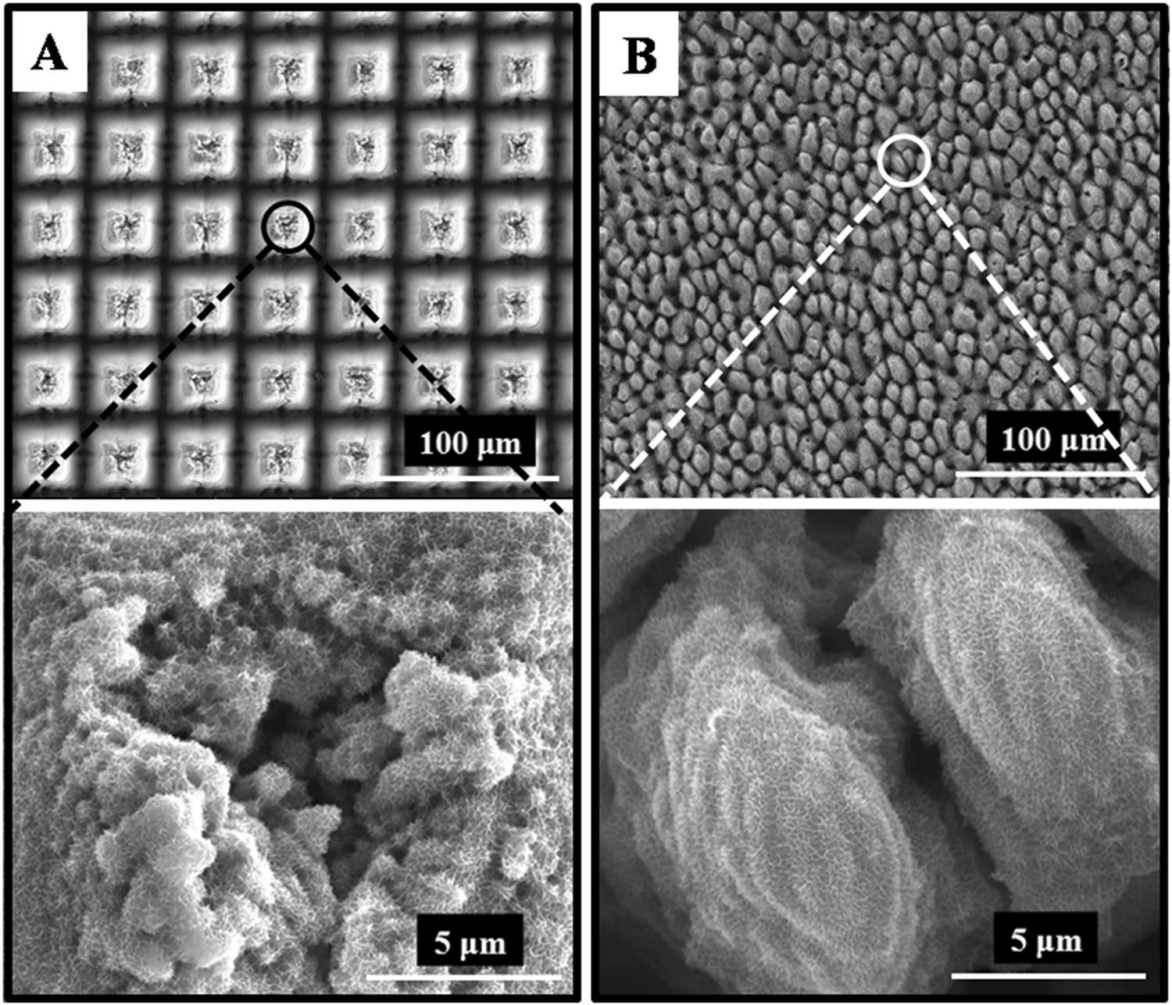
FESEM images of annealing-TiO_2_ with different morphology and size (**A**) Square microarray, and (**B**) Conical microarray.

The annealing-TiO_2_ can be facilely tuned in the micrometer scale by simply controlling the femtosecond laser parameters. Figure 6 shows that the microarray can be fabricated into different morphologies and sizes, while the same nanoporous substructure can be maintained with the same H_2_O_2_ oxidation and annealing conditions. Figure S5A compares the reflectance of annealing-TiO_2_ with square microarray to that of conical microarray. The average reflectance of square microarray was 2–3% lower than that of conical microarray. The photodegradation efficiency increased from 71% of the conical microarray (Figure S5B) to 78% of the square microarray (Figure S5C) accordingly. The results along with Fig. 5 indicated that the functions and properties of the final annealing-TiO_2_ could be enhanced by optimizing the structure. However, a more systematic study is needed to further corroborate our suggestion.

In conclusion, we fabricated substrate-grown micro/nanostructured anataseTiO_2_ with tunable morphology and size by a hybrid method involving femtosecond laser fabrication followed by H_2_O_2_ oxidation and annealing. The fs-Ti fabricated by femtosecond laser had superior light harvesting property in 200–1000 nm wavelength range which was inherited by annealing-TiO_2_, resulting in improved photocatalytic degradation of methyl orange under UV-light irradiation compared to nanoporous-TiO_2_ fabricated through H_2_O_2_ oxidation and annealing only (without laser fabrication). Meanwhile, annealing-TiO_2_ showed good cyclic performance, which promises significance in practical application. Furthermore, as annealing-TiO_2_ was capable of effective capture of visible light, it also holds potential for application in dye-sensitized solar cells. The proposed hybrid fabrication method can be readily extended to fabrication of a wide range of functional metal oxides, which indicates great potential in applications such as solar cells, photocatalysis, and lithium-ion battery.

## Methods

### Materials

Pure Ti sheets (purity 99.99%) were purchased from China New Metal Materials Technology Co., Ltd. H_2_O_2_ solution (30 wt.%) was purchased from Chinese Medicine Group Chemical Reagent Co., Ltd. Methyl orange was purchased from Alfa Aesar (China) Chemicals.

### Fabrication Process

First, the Ti sheets were cut into 20 mm × 20 mm × 1 mm squares, abraded by SiC abrasive sandpaper, and ultrasonically cleaned in acetone for 10 minutes. Then, the Ti squares were scanned with a femtosecond laser (Trumpf TruMicro 5000), which generates laser pulses at central wavelength of 1030 nm, peak power of 80 W, duration of 800 fs, and repetition rate of 400 kHz. A scanning galvanometer was used to control the laser scanning routine and the scanning velocity was 500 mm/s. The different microstructures were fabricated by controlling the laser scanning parameters, which were summarized in Table S1. Next, the samples were ultrasonically cleaned in deionized water for 10 min to remove the metal powder produced during laser processing, and then immersed in 40 mL H_2_O_2_ solution at 80 °C for 1 h. Finally, the samples were rinsed by deionized water and annealed in air at 450 °C for 1 h.

### Characterization and Measurements

The morphology of the samples was examined by LSCM (Keyence VK-X130 K), FESEM (FEI Quanta650), and SEM (Hitachi S-3400N) equipped with EDS. The thin film of annealing-TiO_2_ was observed using TEM (Zeiss Libra 200 FE). To analyze the composition, XRD patterns were recorded by an X-ray diffractometer (Bruker D8 Advance, Cu target). The angle between the sample surface and incident X-ray beam was set at 1° and the detector scanned from 20 to 80°. Surface analysis was carried out by XPS using an X-ray photoelectron spectrometer (PHI 5300) with a monochromatic Al Kα source and a pass energy of 30 eV and a step size of 0.05 eV. The surface reflectance measurements were carried out using an integrating sphere by Shimadzu UV-3600 UV-Vis spectrophotometer. The photocatalytic activity was evaluated using methyl orange solution (initial concentration 10 mg L^−1^). Before irradiation, the samples were immersed in 20 mL methyl orange solution in darkness for 30 min to reach the adsorption-desorption equilibrium. Then, while still immerse in the solution, the samples were irradiated with a 125 W high pressure mercury lamp placed 15 cm away, which emited 365 nm ultraviolet light. Stirring of the solution was maintained throughout the whole process. 1 mL solution was taken out every 40 min during irradiation to measure the absorption spectra within the wavelength range of 250–600 nm by an UV-Vis spectrophotometer (Shimadzu UV-2450). The absorbance at 460 nm was used to calculate the degree of photodegradation of methyl orange at different times. In the durability tests, samples were repeated in ten consecutive cycles of photocatalysis. The samples were washed thoroughly with deionized water and dried after each cycle.

## Electronic supplementary material

Femtosecond Laser Fabrication of Anatase TiO2 Micro-nanostructures with Chemical Oxidation and Annealing
