# Supplementary material for: Femtosecond Laser Fabrication of Anatase TiO2 Micro-nanostructures with Chemical Oxidation and Annealing
Source: Sci Rep. 2017 May 18;7:2089. doi: 10.1038/s41598-017-02369-w (PMC5437094; doi:10.1038/s41598-017-02369-w)
Supplement: Supplementary file 1 — Femtosecond Laser Fabrication of Anatase TiO2 Micro-nanostructures with Chemical Oxidation and Annealing [file 41598_2017_2369_MOESM1_ESM.doc]

**Femtosecond Laser Fabrication of Anatase TiO_2_ Micro-nanostructures with Chemical Oxidation and Annealing**

Ting Huang^*^, Jinlong Lu, Xin Zhang, Rongshi Xiao, Wuxiong Yang, and Qiang Wu

*High-power and Ultrafast Laser Manufacturing Lab, Institute of Laser Engineering, Beijing University of Technology, Beijing, 100124, China*


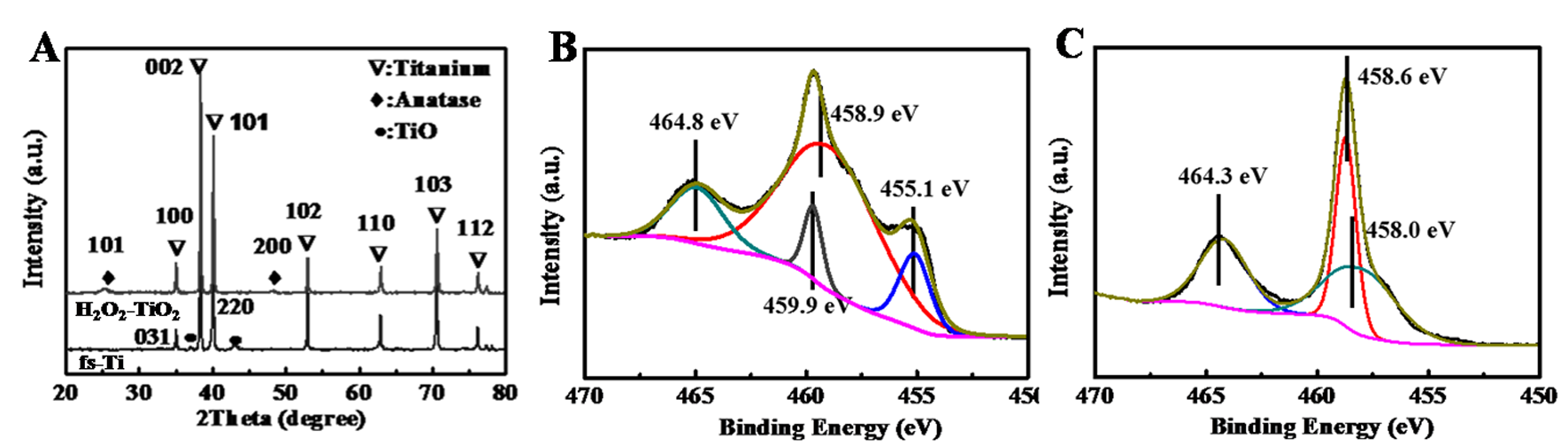


Figure S1. (A) XRD patterns of the structure after laser fabrication (fs-Ti) and H_2_O_2_ oxidation (H_2_O_2_-TiO_2_) and (B) Ti 2p XPS of fs-Ti and H_2_O_2_-TiO_2_.


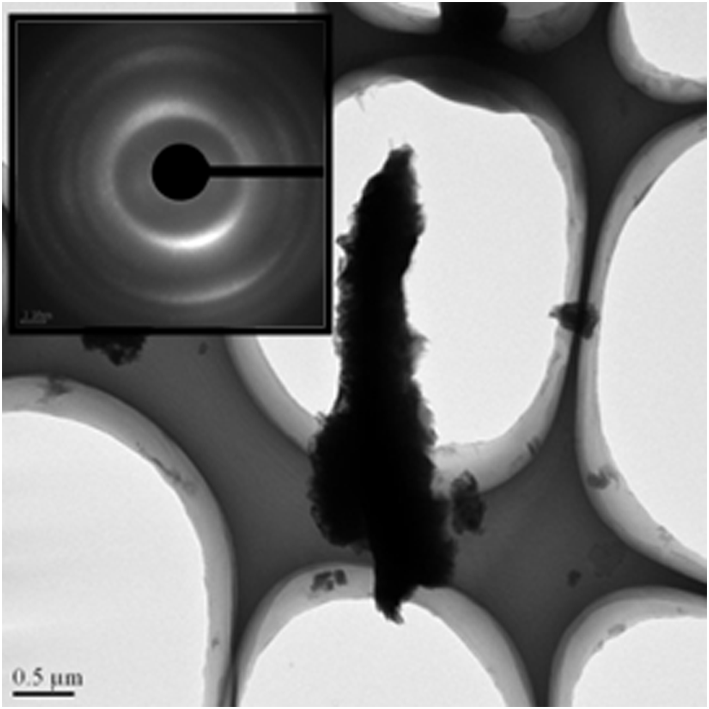


Figure S2. TEM image with inset diffraction pattern showing amorphous phase formed by H_2_O_2_ oxidation.


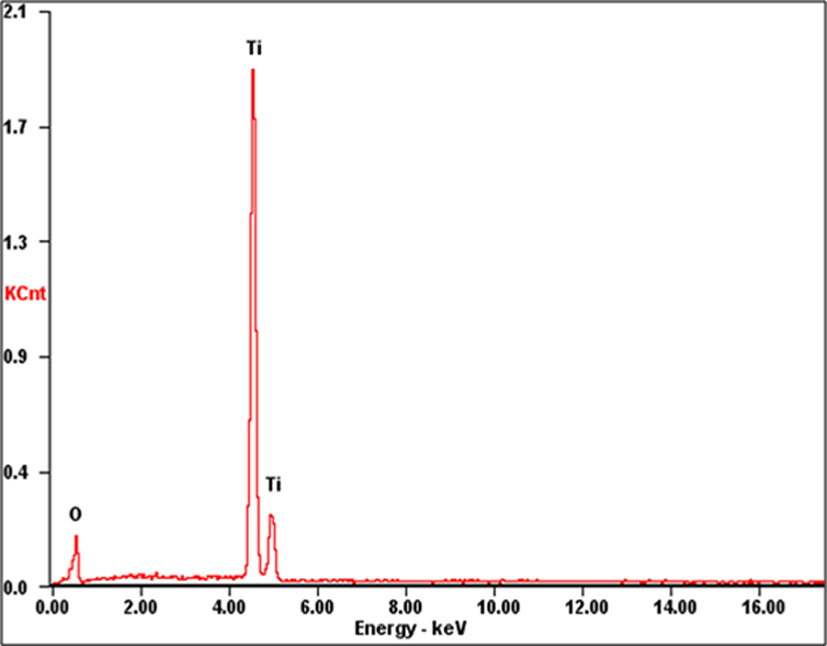


Figure S3. EDS result showing the constituent elements of the final annealing-TiO_2_.


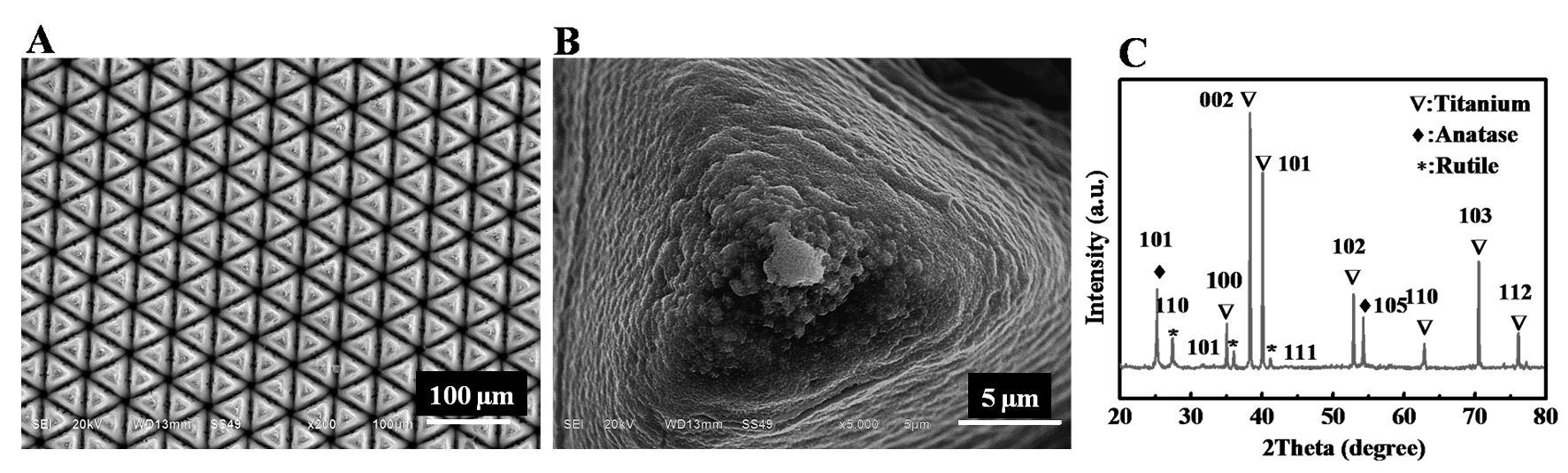


Figure S4. (A) and (B) SEM top-view images at different magnifications, and (C) XRD pattern of annealing-TiO_2_ after the cycling degradation tests.


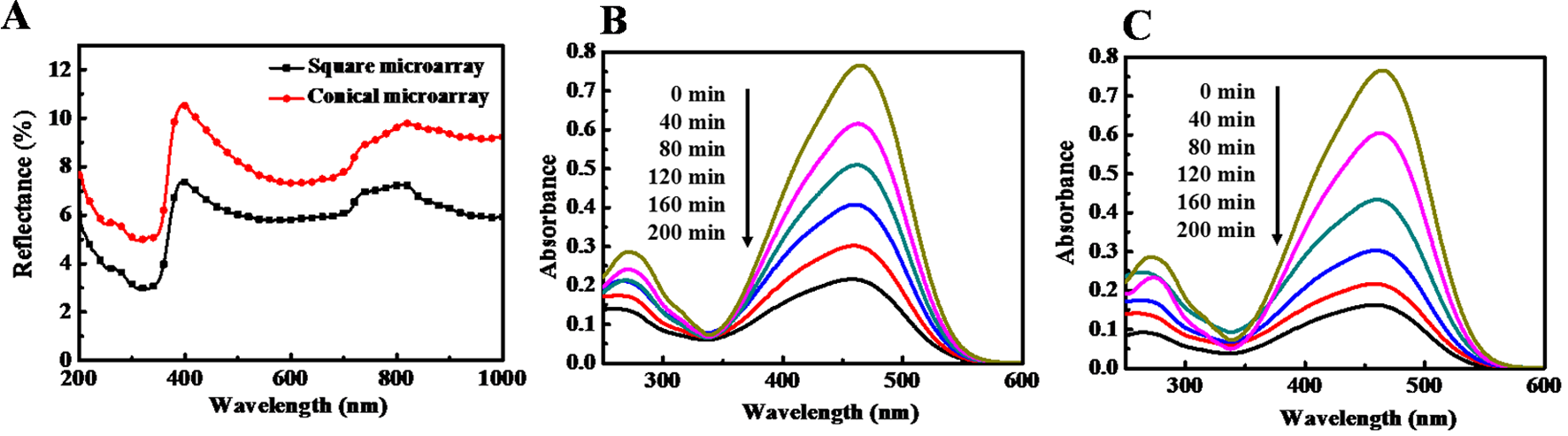


Figure S5. (A) Reflection spectra of annealing-TiO_2_ with square microarray and conical microarray, temporal UV-visible adsorption spectral of annealing-TiO_2_ with (B) Conical microarray and (C) Square microarray.

Table S1 Laser scanning parameters

| Structure | Laser power | Laser spot diameter | Line spacing | Filling angle | Scanning times |
| --- | --- | --- | --- | --- | --- |
| Triangular | 4 W | 40 μm | 45 μm | 60° | 180 |
| Square | 4 W | 40 μm | 40 μm | 90° | 120 |
| Conical | 8 W | 160 μm | 50 μm | 0° | 10 |
